# Plasticity of Neural Systems in Tinnitus

**DOI:** 10.1155/2014/968029

**Published:** 2014-09-08

**Authors:** Martin Meyer, Berthold Langguth, Tobias Kleinjung, Aage R. Møller

**Affiliations:** ^1^University of Zürich, Psychological Institute, Neuroplasticity and Learning in the Healthy Aging Brain, Andreasstraße 15/Box 2, 8050 Zürich, Switzerland; ^2^Department of Psychiatry and Psychotherapy, University of Regensburg, Universitaetsstraße 84, 93053 Regensburg, Germany; ^3^Department of Otorhinolaryngology, University of Zürich, Frauenklinikstraße 24, 8091 Zürich, Switzerland; ^4^School of Behavioral and Brain Sciences, University of Texas at Dallas, 800 W. Campbell Road, Richardson, TX 75080, USA

This special issue of the journal is dedicated to tinnitus and the role of neuroplasticity in its symptomology. In western industrial countries with a steadily aging population, the number of individuals who suffer from tinnitus is immense. Approximately 50 million people in the USA and 70 million individuals in the European Union, that is, approximately 10% of the population, are affected. A fraction of those concerned individuals indicate a significant loss of quality of life. Tinnitus has two main forms: objective and subjective. Objective tinnitus, caused by sounds actually generated in the body, is rare. Subjective tinnitus, the more common form, is a phantom sensation. Tinnitus may be intermittent or constant (chronic) and its strength and its nature may vary. The causes of many forms of tinnitus are unknown and the treatments, therefore, focus on the management of symptoms.

Meanwhile, it is widely accepted that tinnitus must not be conceived as a sole dysfunction of the inner ear. It has rather been agreed that tinnitus emanates from a perplexing network that includes the ear and the auditory pathway but primarily resides in the human brain.

It is generally accepted that people with subjective tinnitus may experience two kinds of symptoms: one is the hearing of a sound that does not come from the environment and the other experience is a form of distress or suffering. These two kinds of symptoms are not directly related and an individual who experiences a weak tinnitus sound may nonetheless experience severe suffering. Others may experience a strong sound but suffer little or not at all. It seems likely that these two expressions of tinnitus have different pathologies and may engage different circuits in the brain.

The key to development of new treatments is a better understanding of the pathology of the disorder. Recent years have seen important progress in the understanding of pertinent aspects of the neuropsychology and neurobiology of subjective idiopathic tinnitus but many questions remain unanswered in that rapidly burgeoning field of neuroscience. The anatomical location of the pathology that causes the phantom sound is not completely known nor is it known what changes in the brain are directly or indirectly associated with distress or suffering. Recent advances in neuroscience and clinical medicine have introduced new models and frameworks that help elucidate the mechanisms underlying the pathology of subjective tinnitus.

Recent studies indicate that changes in connections in many parts of the brain play an important role in causing the symptoms of tinnitus mentioned above. The networks formed by these connections consist of cortical and subcortical areas that serve auditory as well as other functions. Understanding the abnormalities in these networks and their dynamic interactions (connectivity) is of utmost importance for understanding different people's experience of tinnitus. Such knowledge is naturally also important for developing effective treatments of tinnitus and of the associated symptoms of distress and suffering.

Management of idiopathic tinnitus is a challenge and effective treatment options are still limited. The main reason for these obstacles in management of the tinnitus patient is insufficient knowledge and understanding of the pathology of the many forms of tinnitus. The tinnitus patient is a challenge to the physician or neuropsychologist for several reasons. Idiopathic tinnitus is not a single well-defined disease but a series of very different disorders. There are no objective tests that can distinguish between the different forms of tinnitus. This means that the patient's own description is so far the only basis for treatment. In those few forms of tinnitus known to stem from a specific treatable disease, the treatment of that underlying cause is often the best option though tinnitus without a known treatable underlying cause must be alleviated through the management of its symptoms.

This special issue has 14 articles covering the underpinning pathology, diagnosis, and treatments of chronic tinnitus. The articles report results of experimental studies in animals and studies in persons with tinnitus using different forms of imaging and electrophysiological techniques. Psychiatric, neurological, neuropsychological, and otological facets are comprehensively covered and discussed. The papers provide novel approaches for understanding the pathology and accordingly potential treatment of many forms of tinnitus. Some of the articles discuss abnormalities in EEG-based connectivity and others discuss the changes in the brain after specific treatments for tinnitus. Several of the articles in the special issue provide critical analysis of the efficacy of different forms of treatment of tinnitus while one of the articles discusses the psychiatric comorbidity of tinnitus.

We are convinced that this compilation of inspiring papers will be evidently well received as a crucial step towards better dealing with chronic tinnitus and we are delighted to introduce this special issue to the readers.

*Martin Meyer* *Martin Meyer* *Berthold Langguth* *Berthold Langguth* *Tobias Kleinjung* *Tobias Kleinjung* *Aage R. Møller* *Aage R. Møller*

